# Inverse Regulation of Cartilage Neogenesis at Physiologically Relevant Calcium Conditions by Human Articular Chondrocytes and Mesenchymal Stromal Cells

**DOI:** 10.3390/cells12121659

**Published:** 2023-06-18

**Authors:** Tim Hammersen, Justyna Buchert, Severin Zietzschmann, Solvig Diederichs, Wiltrud Richter

**Affiliations:** 1 Research Center for Experimental Orthopaedics, Department of Orthopaedics, Heidelberg University Hospital, 69118 Heidelberg, Germany; 2 Orthopaedic Hospital, Department of Orthopaedics, Heidelberg University Hospital, 69118 Heidelberg, Germany

**Keywords:** extracellular matrix, tissue engineering, parathyroid hormone-related peptide, stem cells, chondrogenesis

## Abstract

Elaborate bioreactor cultivation or expensive growth factor supplementation can enhance extracellular matrix production in engineered neocartilage to provide sufficient mechanical resistance. We here investigated whether raising extracellular calcium levels in chondrogenic cultures to physiologically relevant levels would provide a simple and inexpensive alternative to enhance cartilage neogenesis from human articular chondrocytes (AC) or bone marrow-derived mesenchymal stromal cells (BMSC). Interestingly, AC and BMSC-derived chondrocytes showed an opposite response to a calcium increase from 1.8 mM to 8 mM by which glycosaminoglycan (GAG) and collagen type II production were elevated during BMSC chondrogenesis but depressed in AC, leading to two-fold higher GAG/DNA values in BMSC-based neocartilage compared to the AC group. According to control treatments with Mg^2+^ or sucrose, these effects were specific for CaCl_2_ rather than divalent cations or osmolarity. Importantly, undesired pro-hypertrophic traits were not stimulated by calcium treatment. Specific induction of PTHrP mRNA and protein by 8.0mM calcium only in AC, along with negative effects of recombinant PTHrP_1-34_ on cartilage matrix production, suggested that the PTHrP pathway contributed to the detrimental effects in AC-based neocartilage. Altogether, raising extracellular calcium levels was discovered as a novel, simple and inexpensive stimulator for BMSC-based cartilage neogenesis without the need for special bioreactors, whereas such conditions should be avoided for AC.

## 1. Introduction

The unique viscoelastic and compressive properties of cartilage tissue are provided by the extracellular matrix (ECM) which is synthesized by chondrocytes and mainly composed of proteoglycans, glycosaminoglycans (GAG) and collagen type II. Although chondrocytes are metabolically very active, they do not divide after adolescence in vivo and this limits regeneration of cartilage to very small defects associated with minimal loss of matrix components. Larger damage exceeds the intrinsic repair capacity of cartilage, becomes permanent and can lead to progressive degeneration and development of osteoarthritis [1].

Modern tissue engineering-based cartilage repair strategies aim at the full restoration of tissue function by replacing lost chondrocytes and the unique ECM which determines the mechanical integrity of articular cartilage [2]. Autogenous human articular chondrocytes (AC) or mesenchymal stromal cells (MSC) are expanded and differentiated in vitro to form mature neocartilage with high ECM content which can resist the challenging mechanical conditions in the joint. Lack of cartilage matrix makes implants mechanically inferior to native tissue and therefore prone to failure [3,4]. Current engineered cartilage [5,6] or matrix-assisted in vivo repair tissue [7,8] still provides inferior mechanical resilience compared to native cartilage, limiting long-term resistance to mechanical wear. Since mechanical stiffness of neocartilage directly correlates with its GAG [5,6,7,9] and collagen content [7,9], the deposition of these ECM components should be high to gain fully functional neocartilage. ECM content of engineered cartilage was already enhanced via a higher cell seeding density [9], by prolongation of the culture period [9,10], application of long-term mechanical stimulation [11,12] or physioxia in bioreactors [13,14] or by additional growth factor supplementation [5,15,16]. Nevertheless, in order to avoid the use of elaborative bioreactors or expensive growth factor treatments, a cheaper and easily applicable method is desired to reproducibly enhance the ECM content of cartilage replacement tissue.

Physiologically, extracellular calcium ions (Ca^2+^) are central for the structure and function of cartilage ECM by electrostatically binding to proteoglycans. The chelation of divalent ions from cartilage explants using EDTA or EGTA induced severe proteoglycan degradation, which was rescued by the simultaneous addition of 5 mM CaCl_2_ [17]. Furthermore, a raise in extracellular calcium concentration from 1.8 mM to 5 mM or 10 mM was suggested as an important inducer of matrix metabolism during cartilage development [18,19]. Importantly, the range of physiologically relevant extracellular Ca^2+^ concentration ([Ca^2+^]_e_) in native articular cartilage increases with the depth of the tissue from 6 to 20 mM [20]. Intriguingly, Ca^2+^ conditions so far applied during in vitro cartilage neogenesis were hypo-physiological since studies were conducted with 1.8 mM calcium contained in standard culture media. Thus, current neocartilage had to mature under considerably lower calcium levels than native cartilage and surprisingly little is known so far about the effect of physiologically relevant calcium conditions on cartilage matrix deposition by AC and MSC.

Monolayer and suspension culture models with AC were performed with elevated calcium but were not informative since no 3D cartilage tissue was formed [18,19,21]. In the only 3D culture study under physiologically relevant extracellular calcium conditions (8.0 mM [Ca^2+^]_e_), AC from 4-week-old calves were applied in alginate gel, and significantly less glycosaminoglycan (GAG) at unchanged total collagen content was observed compared to 1.8 mM [Ca^2+^]_e_ controls after 28 days [22]. However, human adult AC substantially differ from juvenile bovine AC in many aspects, including their response to pro-chondrogenic stimuli [23,24]. Hence, the potential of physiologically relevant calcium levels to promote human cartilage neogenesis from AC in vitro is largely unknown.

Unlike AC, MSC undergo endochondral differentiation into hypertrophic chondrocytes in vitro, and MSC-based neocartilage can mineralize and develop into bone in vivo [25,26]. Two studies investigated the effect of elevated extracellular calcium levels on chondrogenesis of human chondroprogenitor cells in 3D culture. Both differentiated the MSC in the presence of specifically pro-hypertrophic factors such as L-thyroxine or BMP6 which is undesired for articular cartilage neogenesis. Under pro-hypertrophic conditions and 8.0 mM [Ca^2+^]_e_, human adipose tissue-derived MSC [27] as well as human bone marrow-derived MSC (BMSC) [28] deposited less GAG and collagen type II compared to control cultures. Furthermore, increased deposition of hypertrophic collagen type X protein as well as in vitro mineral deposition were observed [27]. This indicated that calcium may enhance chondrocyte hypertrophy which is undesirable for cartilage regeneration therapy. Importantly, in consideration of the well-documented interactions of thyroxine with calcium [29,30] and crosstalk between thyroxine and BMPs with calcium signaling pathways, their presence may have significantly influenced the calcium effects [31,32]. Our own data on chondrogenesis of human BMSC performed in the absence of pro-hypertrophic factors indicated that cells differentiated better in the presence of a resorbable β-tri-calcium-phosphate (TCP) than in its absence [33]. Thus, the hypothesis was raised that calcium-release from resorbable β-TCP may have enhanced cartilage formation. In this light, a study on the potential of physiologically relevant extracellular calcium levels to promote cartilage neogenesis appeared promising under standard chondrogenic conditions with BMSC as a cell source.

The objective of this study was to elucidate whether raising the extracellular calcium concentration in chondrogenic differentiation culture to physiologically relevant levels would promote cartilage neogenesis from human AC or BMSC. Our main goal on the path to fully functional neocartilage was to increase the proteoglycan and collagen type II content of constructs without a stimulation of unwanted pro-hypertrophic side effects and to elucidate relevant signaling pathways for calcium-related effects. A well characterized engineered cartilage model based on primary human AC or BMSC seeded into collagen type I/III carriers and cultured under chondrogenic conditions was applied [5,34,35,36,37]. After 35 days of maturation either under standard conditions or under physiologically relevant 8.0 mM [Ca^2+^]_e_, the engineered tissue was characterized for the expression of chondrocyte markers, the biosynthesis of GAGs, the content of GAG/DNA and the total deposition of collagen type II protein. Hypertrophy and osteogenesis markers were analyzed at a gene and protein level to determine effects on the chondrocyte phenotype. Relevant signaling mechanisms of calcium-related effects were judged by quantifying prominent pro- and anti-chondrogenic molecules SOX9, TGFβ, BMP and PTHrP on gene, protein and pathway activity level.

## 2. Materials and Methods

### 2.1. Isolation and Expansion of AC and MSC

Samples of human articular cartilage were obtained from 11 patients (5 female and 6 male, age range 55 to 87, mean age 65 ± 10 years) undergoing total knee replacement surgery. Human bone marrow aspirates were obtained from 9 patients (5 female and 4 male, age range 53 to 83, mean age 64 ± 10 years) undergoing total hip replacement. The study was approved by the local ethics committee on human experimentation of the Medical Faculty of Heidelberg and performed in agreement with the Helsinki Declaration of 1975 in its latest version. Written informed consent was obtained from all individuals included in the study.

Human AC were isolated from regions with no evident degeneration as described previously [38]. In brief, cartilage was minced and digested for 16 h at 37 °C in 1.5 mg/mL collagenase B (Roche, Mannheim, Germany) and 0.1 mg/mL hyaluronidase (Merck, Darmstadt, Germany). Chondrocytes were washed and filtered to obtain a single cell suspension. Cells were plated at 5700 cells/cm^2^ and expanded for 2 passages in low glucose-containing Dulbecco’s modified Eagle’s medium (DMEM, Gibco, Life Technologies, Darmstadt, Germany) supplemented with 10% fetal bovine serum (FBS, Sigma-Aldrich, Darmstadt, Germany) and 1% penicillin/streptomycin (Biochrom, Darmstadt, Germany) at 37 °C, 6% CO_2_.

Human BMSCs were obtained from bone marrow aspirates, as described previously [39]. In brief, the mononuclear cell fraction was separated from bone marrow aspirates by Ficoll Paque Plus (Cytiva, Freiburg, Germany) density gradient centrifugation. Cells were washed with phosphate-buffered saline (PBS), seeded into 0.1% gelatin-coated culture flasks at a density of 5000 cells/cm^2^ and cultured in expansion medium (DMEM high glucose, 12.5% FBS, 2 mM L-glutamine, 1% non-essential amino acids, 50 μM 2-mercapthoethanol (all from Gibco, Life Technologies, Darmstadt, Germany), 1% penicillin/streptomycin (Biochrom, Darmstadt, Germany) and 4 ng/mL basic fibroblast growth factor (bFGF, Active Bioscience, Hamburg, Germany)) at 37 °C, 6% CO_2_. After 24 h, non-adherent cells were removed by washing with PBS and the attached cells were expanded for 3 passages.

### 2.2. Maturation and Stimulation of Engineered Cartilage

AC or BMSC were seeded on both sides of a type I/III collagen carrier (Optimaix^®^, Matricel GmbH, Herzogenrath, Germany; 4 mm diameter, 1.5 mm height) at 5 × 10^5^ cells per construct and cultured in chondrocyte differentiation medium as described [5]. Chondrogenic medium was DMEM high glucose (Gibco, Life Technologies, Darmstadt, Germany), 0.1 μM dexamethasone, 0.17 mM ascorbic acid 2-phosphate, 5 μg/mL transferrin, 5 ng/mL sodium selenite, 5 mM sodium pyruvate, 0.35 mM proline, 1.25 mg/mL BSA (all from Sigma-Aldrich, Darmstadt, Germany), 1% penicillin/streptomycin (Biochrom, Darmstadt, Germany), 5 μg/mL insulin (Sanofi-Aventis, Frankfurt am Main, Germany) and 10 ng/mL TGF-β1 (Biomol, Hamburg, Germany) at 37 °C, 6% CO_2_. For BMSC, insulin, transferrin, sodium selenite and BSA were replaced by ITS+ premix (Corning, New York City, NY, USA), leading to similar supplement concentrations as used for AC. The medium was changed three times a week.

For calcium treatment from day 0 to culture termination, CaCl_2_ was added to a final concentration of 8.0 mM, based on the range of calcium concentration in native articular cartilage [20,40], as well as past studies, to investigate calcium effects in AC and MSC-derived chondrocytes [22,27,28]. For PTHrP stimulation, control constructs were treated from day 21 to day 35 with 10 ng/mL human recombinant PTHrP_1-34_ (Bachem, Bubendorf, Switzerland).

### 2.3. Glycosaminoglycan and DNA Quantification

For determination of the total GAG and DNA content, constructs were digested in 1 mL Tris-HCl buffer with 0.5 mg/mL Proteinase-K. Digestion was performed at 60 °C overnight while shaking at 800 rpm. The GAG content within the digests was quantified using the 1,9-dimethylmethylene blue (DMMB) assay according to [41] with values deduced from a chondroitin-6-sulfate standard curve. The GAG content of the sample was normalized to its DNA content determined by Quant-iT™-PicoGreen^®^ kit (Invitrogen, Darmstadt, Germany) according to the manufacturer’s instructions.

### 2.4. Radiolabel Incorporation

GAG synthesis was measured, as described before [34], during the last 24 h before termination of culture on day 35. Engineered cartilage was placed on a nylon mesh in a 48-well plate to allow labeling from all sides. Samples were labeled with 4 μCi ^35^S as Na_2_SO_4_ (Hartmann Analytic, Braunschweig, Germany) in 500 µL of chondrocyte differentiation medium with or without the addition of CaCl_2_ or PTHrP_1-34_. After 5 washing steps with unlabeled 1 mM Na_2_SO_4_ in PBS for 20 min while shaking, samples were digested in 1 mL of Tris-HCl buffer with 0.5 mg/mL proteinase-K at 60 °C and 800 rpm overnight. The incorporated label was quantified by β-scintillation counting using the program Winspectral, version 1414. Radioactive count was normalized to the DNA content of the same lysate.

### 2.5. Total RNA Isolation and mRNA Expression Analysis

Samples were harvested and quick-frozen in liquid nitrogen. As previously described [35], total RNA was isolated from disintegrated samples by a phenol/guanidine isothiocyanate extraction (peqGOLD TriFast™, Peqlab, Erlangen, Germany) and purified using Zymoclean™ Gel DNA Recovery Kit (ZymoResearch, Irvine, CA, USA) according to the manufacturer’s instructions. cDNA was reverse transcribed from 500 ng of total RNA using oligo(dT) primers and Omniscript reverse transcriptase (Qiagen, Hilden, Germany). Alterations in gene expression were monitored via detection of Absolute qPCR SYBR-Green-Mix (Life Technologies, Darmstadt, Germany) in a LightCycler^®^ 96 system (Roche, Mannheim, Germany). Gene expression percentages of reference genes were calculated as 1.8^−ΔCt^ as recommended by the manufacturer of the PCR cycler. ΔCt is the difference between Ct values of the gene of interest and the arithmetic mean of the reference genes *18S*, *RPL13* and *GAPDH*. The PCR products were quality-checked by agarose gel electrophoresis and examination of melting curves. Primer sequences are summarized in Supplementary Table S1.

### 2.6. Histology and Immunohistochemistry

Constructs were fixed in 4% paraformaldehyde for 4 h, dehydrated in a graded 2-propanol series, embedded in paraffin and microsectioned in 5 μm sections. After rehydration, proteoglycan deposition was analyzed by staining sections with 0.2% (*w*/*v*) Safranin O (Fluka, Sigma-Aldrich, Darmstadt, Germany) in 1% acetic acid and counterstaining with 0.04% (*w*/*v*) Certistain Fast Green (Merck, Darmstadt, Germany) in 0.2% acetic acid.

Immunohistological staining against type II collagen was performed as described previously [39]. Briefly, 5 µm sections were incubated with hyaluronidase (4 mg/mL) in PBS (pH 5.5) and pronase (1 mg/mL, both from Roche Diagnostics, Mannheim, Germany). Sections were blocked with 5% bovine serum albumin (BSA, Sigma-Aldrich, Darmstadt, Germany) and incubated with mouse anti-human type II collagen antibody (II-4C11; 1:1000, ICN Biomedicals, Irvine, CA, USA). Detection was performed using biotinylated goat anti-mouse secondary antibody, streptavidin alkaline phosphatase (30 min, 20 °C, Dako, Glostrup, Denmark) and fast red (Sigma-Aldrich, Darmstadt, Germany).

### 2.7. Western Blotting

Constructs were harvested and quick-frozen in liquid nitrogen. Proteins were extracted in PhosphoSafe™ Extraction-Reagent (Merck, Darmstadt, Germany) containing 1% Pefabloc^®^ SC (Sigma-Aldrich, Darmstadt, Germany) in a mixer mill (Retsch, Haan, Germany) at 30 Hz for 2 × 2 min with intermediate cooling on ice for one minute. Cellular debris were removed from lysate by centrifuging the samples for 20 min at 13,000× *g* and 4 °C. 30 μg of protein were separated by denaturing sodium-dodecyl sulfate polyacrylamide gel electrophoresis and blotted on a nitrocellulose membrane (GE Healthcare, Amersham, Solingen, Germany) following standard protocols [37]. When applicable, the membrane was cut horizontally in order to detect several proteins of interest in the same lysates. The membranes were probed with mouse anti-β-actin antibody (clone AC-15; 1:10,000; GeneTex; GTX26276), rabbit monoclonal anti-pSMAD1/5/9 antibody (1:250; Cell Signaling Technology; 13820), rabbit monoclonal SMAD1/5 antibody (1:500, 1:1000; abcam; ab33902, ab40771), rabbit polyclonal anti-SOX9 antibody (1:2000; Merck Millipore; AB5535), rabbit monoclonal anti-pSMAD2 antibody (1:250; Cell Signaling Technology; 3108) and rabbit monoclonal anti-SMAD2/3 antibody (1:250; Cell Signaling Technology; 8685). Bands were detected by HRP-conjugated goat-anti-mouse-IgG (1:10,000) or HRP-conjugated goat-anti-rabbit-IgG (1:5000; both Jackson ImmunoResearch, Cambridge, UK) and visualized using Lumi-Light Western Blotting substrate (Roche, Mannheim, Germany) or WesternBright ECL-HRP (Advansta, Menlo Park, CA, USA) substrate.

For Western blot detection of collagen type II and collagen type X, collagens were isolated using the pepsin isolation method according to [26]. In brief, constructs were digested in pepsin solution (2.5 mg/mL pepsin in 0.5 M acetic acid, 0.2 M NaCl pepsin buffer) for at least 16 h at room temperature to degrade all proteins except collagens. The pH was then adjusted to neutral pH 7 with 1 M Tris Base followed by extraction of collagens with 4.5 M NaCl overnight at 4 °C. After centrifuging, the pellets were resuspended in 400 μL precipitation buffer (0.1 M Tris Base, 0.4 M NaCl) and the collagens were precipitated for 4 h at −20 °C with 100% ethanol. After centrifugation, the collagen pellets were resuspended in lysis buffer (50 mM Tris, 150 mM NaCl, 1% Triton X-100). For analysis, samples were subjected to Western blot analysis as described above. The lower part of the membrane was incubated with mouse anti-human type X collagen antibody (X53; 1:500; Quartett, Berlin, Germany) and the upper part with mouse anti-human type II collagen antibody (II-4C11; 1:1000; ICN Biomedicals, Irvine, CA, USA) at 4 °C overnight and detected by HRP-conjugated goat-anti-mouse- IgG (1:10,000; Jackson ImmunoResearch, Cambridge, UK).

### 2.8. ALP Enzyme Activity

Culture supernatants (pooled from 2 replicate constructs per group), conditioned for 48 h, were collected and incubated with substrate solution (10 mg/mL of p-nitrophenyl phosphate in 0.1 M glycine, 1 mM MgCl_2_ and 1 mM ZnCl_2_, pH 9.6) and ALP activity was measured after 120 min spectrophotometrically at 405/490 nm (FLUOStar Omega, BMG LABTECH, Offenburg, Germany). The substrate conversion was referred to a p-nitrophenol-derived standard curve (Sigma-Aldrich, Darmstadt, Germany) and calculated as ALP enzyme activity (ng/mL/min).

### 2.9. PTHrP Protein Quantification

Constructs were harvested and quick-frozen in liquid nitrogen and proteins were isolated as described above. To remove traces of extraction reagent, proteins were precipitated with ice-cold acetone for 15 min, followed by centrifugation and resuspension of the protein pellet in pooled PTHrP-negative normal human blood plasma. Measurement of PTHrP protein levels was performed by Laboratory Dr. Limbach (Heidelberg, Germany) using the active PTHrP immunoradiometric assay method (Immunotech, Prague, Czech Republic).

### 2.10. Statistical Analysis

The number of independent experiments performed for each analysis is given in the figure captions. The data are depicted as boxplots, with each box representing the interquartile range (IQR) extending between the 25th and 75th percentiles, and lines inside the boxes representing the median. Whiskers extend to minimum and maximum values. Outliers were determined according to Tukey method (>1.5 × IQR) and depicted as filled circles. In the case of time course experiments, data are expressed as the mean ± standard error of the mean. Statistical significance between two groups was calculated using Mann–Whitney U test (MWU). A *p* value below 0.05 was considered statistically significant. The data were analyzed using SPSS-25 (IBM, Armonk, NY, USA).

## 3. Results

### 3.1. Inverse Regulation of Cartilage Formation by AC and BMSC-Derived Chondrocytes at Physiologically Relevant Extracellular Calcium Levels

**Figure 1:**
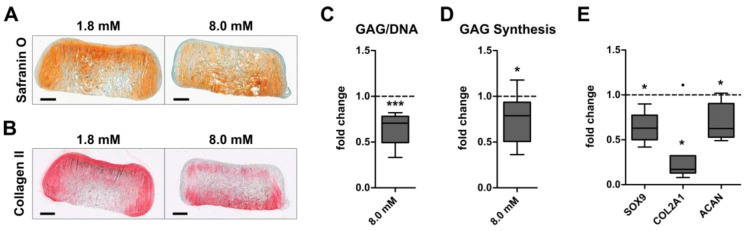
Calcium-induced regulation of cartilage matrix production in AC. A total of 5 × 10^5^ human articular chondrocytes (AC) were seeded in a type I/III collagen carrier and subjected to chondrocyte redifferentiation medium for 35 days at control (1.8 mM) or physiologically relevant (8.0 mM) extracellular [Ca^2+^]_e_. (**A**) Paraffin sections stained with SafraninO/Fast green visualize GAG deposition. Representative images are shown for AC from *n* = 10 donors. Scale bar: 500 µm. (**B**) Collagen type II deposition was detected by immunohistochemistry. Representative images are depicted from *n* = 6 donors. Scale bar: 500 µm. (**C**) The GAG content of samples was measured by DMMB assay (*n* = 11 from 10 donors) and values were normalized to the DNA content; 1.8 mM [Ca^2+^]_e_ control samples were set to one (dashed line at 1.0). (**D**) GAG synthesis rate was measured as ^35^S-sulfate incorporation during the final 24 h of culture on day 35 (*n* = 9 donors). Values were normalized to the DNA content and 1.8 mM [Ca^2+^]_e_ control samples were set to one (dashed line). (**E**) *COL2A1*, *ACAN* and *SOX9* mRNA levels were determined on day 35 using qPCR. Gene expression levels were normalized to the mean expression of reference genes *18S*, *GAPDH* and *RPL13,* and 1.8 mM [Ca^2+^]_e_ control samples were set to one (*n* = 5–7 donors). Data are shown as box plots with each box representing the interquartile range (IQR) extending between the 25th and 75th percentiles and lines inside the boxes representing the median. Whiskers extend to a maximum of 1.5 IQR and an outlier is depicted as a filled circle. * *p* < 0.05, *** *p* < 0.001, Mann–Whitney U test (MWU), vs. 1.8 mM Ca^2+^.

**Figure 2:**
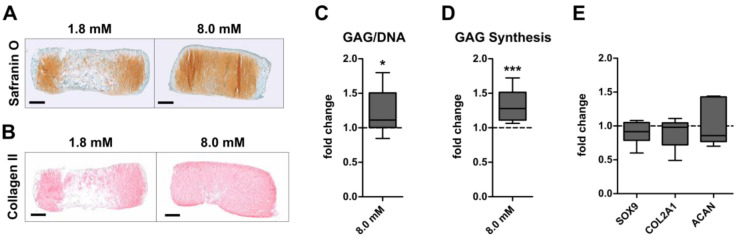
Calcium-induced regulation of cartilage matrix production in BMSC. A total of 5 × 10^5^ human BMSC were seeded in a type I/III collagen carrier and subjected to differentiation medium for 35 days at control (1.8 mM) or physiologically relevant (8.0 mM) extracellular [Ca^2+^]_e_. (**A**) Paraffin sections stained with SafraninO/Fast green visualize GAG deposition. Representative images are depicted for BMSC from *n* = 9 donors. Scale bar: 500 µm. (**B**) Collagen type II deposition was detected by immunohistochemistry. Representative images are depicted for BMSC from *n* = 7 donors. Scale bar: 500 µm. (**C**) GAG content measured by DMMB assay on day 35 (*n* = 10 constructs from 9 donors). Values were normalized to the DNA content, and 1.8 mM [Ca^2+^]_e_ control samples were set to one (dashed line). (**D**) GAG synthesis rate was measured as ^35^S-sulfate incorporation during the final 24 h of culture on day 35 (*n* = 8 donors). Values were normalized to the DNA content and 1.8 mM [Ca^2+^]_e_ control samples were set to one. (**E**) *COL2A1*, *ACAN* and *SOX9* mRNA levels were determined on day 35 using qPCR. Gene expression levels were normalized to the mean expression of reference genes *18S*, *GAPDH* and *RPL13* and 1.8 mM [Ca^2+^]_e_ control samples were set to one (dashed line) (*n* = 6–7 donors). Data are shown as box plots as indicated in Figure 1. * *p* < 0.05, *** *p* < 0.001, MWU, vs. 1.8 mM Ca^2+^.

To investigate whether physiologically relevant extracellular calcium levels can enhance the GAG and collagen type II production of AC or BMSC, engineered neocartilage was matured under chondrogenic conditions at control (1.8 mM) or physiologically relevant (8.0 mM) calcium levels. Histology revealed a cartilage-typical GAG and collagen type II deposition in AC- (Figure 1A,B) and BMSC constructs (Figure 2A,B) under both conditions after 35 days. While 8.0 mM [Ca^2+^]_e_ slightly reduced the staining intensity for proteoglycans (Figure 1A) and collagen type II (Figure 1B) compared to controls in the AC group, this was opposite in BMSC-derived neocartilage (Figure 2A,B). Quantitative analysis of the GAG/DNA content showed that calcium treatment significantly reduced the GAG/DNA-ratio (*p* = 0.001) compared to controls in the AC-group (Figure 1C). This impaired GAG deposition (−30%) at 8.0 mM [Ca^2+^]_e_ was accompanied by a significantly diminished GAG synthesis rate of AC, measured by radiolabel incorporation over the final 24 h of culture on day 35 (−21%, *p* = 0.04; Figure 1D). Likewise, calcium stimulation significantly decreased the expression of the pro-chondrogenic transcription factor *SOX9* and its transcriptional targets *COL2A1* and *ACAN* (Figure 1E). Thus, physiologically relevant extracellular calcium compromised the cartilage matrix formation by AC. In contrast, 8.0 mM [Ca^2+^]_e_ significantly increased the median GAG/DNA content in the BMSC group by 24% (*p* = 0.023, Figure 2C). Of note, while high calcium increased GAG/DNA values compared to controls by up to 85% in 7 out of 10 donor populations, others showed no (*n* = 2) or a slightly reducing (*n* = 1) effect. In addition, a significant increase in the GAG synthesis rate in BMSC constructs was recorded donor-independently on day 35 (+33%, *p* < 0.001, Figure 2D). However, calcium treatment had no effect on the mRNA levels of *SOX9*, *COL2A1* and *ACAN* in BMSC-derived chondrocytes (Figure 2E). Thus, GAG formation was inversely regulated by physiologically relevant calcium levels in AC- and BMSC-derived neocartilage.

### 3.2. Ionic Versus Osmotic Effects on GAG Synthesis of AC- and BMSC-Derived Neocartilage

**Figure 3:**
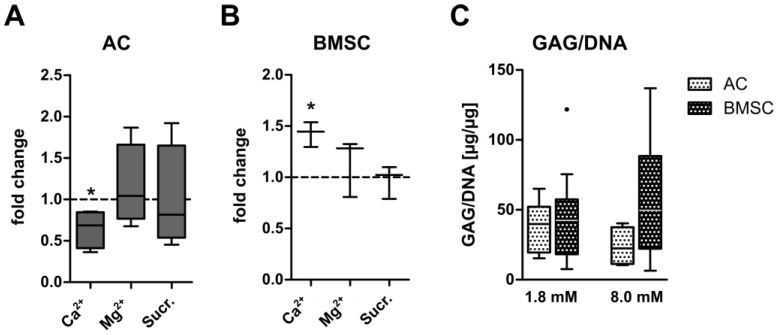
Regulation of GAG synthesis after ionic and osmotic stimulation in AC and BMSC. AC- or BMSC-laden tissue engineering constructs were cultured in differentiation medium for 35 days. In the calcium group, medium calcium concentration was adjusted to 8.0 mM [Ca^2+^]_e_ by the addition of CaCl_2_. Equimolar addition of MgCl_2_ was performed as ionic control in the magnesium group. An equiosmolar concentration of sucrose was added as osmotic control in the sucrose group. GAG synthesis rate was measured as ^35^S-sulfate incorporation during the final 24 h of culture on day 35 in (**A**) AC or (**B**) BMSC. Values were normalized to the DNA content and 1.8 mM [Ca^2+^]_e_ control samples were set to one. Data are shown as box plots as indicated in Figure 1 for samples from *n* = 4 AC- and *n* = 3 MSC donors. * *p* < 0.05, MWU, vs. 1.8 mM Ca^2+^. (**C**) AC- or BMSC constructs were cultured in differentiation medium for 35 days at 1.8 mM or 8.0 mM extracellular [Ca^2+^]_e_. GAG content was measured by DMMB assay on day 35 and normalized to the DNA content in samples from *n* = 10 AC- and *n* = 9 MSC donors. Data are shown as box plots as indicated in Figure 1, MWU, AC vs. BMSC.

In order to determine whether the inverse regulation of GAG synthesis by calcium in AC versus BMSC-derived chondrocytes was a general effect of divalent cations or due to enhanced osmotic conditions, experiments were repeated using 8 mM MgCl_2_ or a corresponding rise in osmolarity of +24 mOsm sucrose as control groups. Importantly, no change in GAG synthesis was observed when AC- or BMSC constructs were cultured for 35 days in the presence of MgCl_2_ or sucrose as ionic or osmotic controls, respectively (Figure 3A,B). Thus, the inverse regulation of GAG synthesis in AC and BMSC-derived chondrocytes was specific to CaCl_2_-treatment. In order to compare the deposition of GAG under physiologically relevant calcium levels between AC and BMSC, GAG/DNA data were subjected to a comparative statistical analysis. While AC and BMSC-derived chondrocytes deposited similar amounts of GAG/DNA at control conditions (*p* = 0.97), the median GAG/DNA content was 2.2-fold higher in BMSC constructs cultured at 8.0 mM [Ca^2+^]_e_ compared to calcium-stimulated AC (Figure 3C). However, due to a large standard deviation between BMSC donor cell populations this just failed significance (*p* = 0.07). Compared to AC cultured under control conditions, calcium stimulation by trend enhanced the median GAG/DNA content in the BMSC group (+25%, *p* = 0.40). Thus, BMSC-derived chondrocytes showed a robust cartilage matrix production under physiologically relevant calcium conditions, depositing about twice the amount of GAG/DNA than AC under identical conditions. Collectively this demonstrates that 8.0 mM extracellular CaCl_2_ treatment provides a simple and inexpensive means of enhancing the ECM content of BMSC-based neocartilage.

### 3.3. No Unwanted Hypertrophic Side Effects by Physiologically Relevant Extracellular Calcium Levels

**Figure 4:**
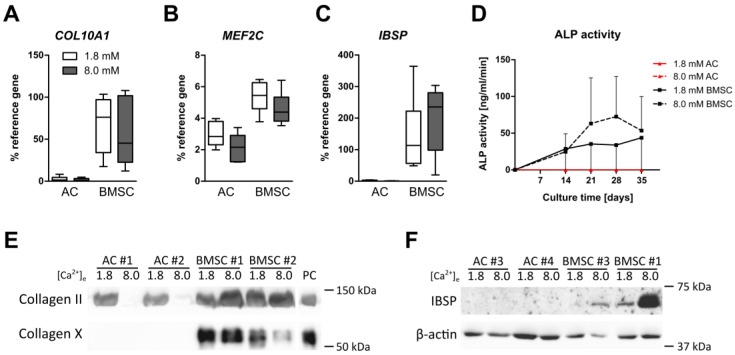
Calcium-induced regulation of endochondral differentiation markers in AC and BMSC-derived neocartilage. AC- or BMSC-laden tissue engineering constructs were cultured in differentiation medium for 35 days at 1.8 or 8.0 mM extracellular [Ca^2+^]_e_. (**A**–**C**) *COL10A1*, *MEF2C* and *IBSP* mRNA levels were determined by qPCR. Gene expression levels were normalized to the mean expression of reference genes *18S*, *GAPDH* and *RPL13*. All data are shown as box plots as indicated in Figure 1. *n* = 6 AC- and *n* = 6 BMSC donors. MWU vs. 1.8 mM Ca^2+^. (**D**) Alkaline phosphatase activity was determined in weekly intervals in pooled culture supernatants from AC or BMSC-derived samples. Data are depicted as median ± 95% confidence interval of *n* = 5 AC- and *n* = 6 BMSC donors. (**E**) Immunoblotting against collagen type II and collagen type X was performed on pepsin digests from AC and BMSC-derived neocartilage on day 35. Representative results are shown for samples from *n* = 2 AC- and *n* = 4 BMSC donors. Pepsin digest from day 42 BMSC micromass pellets served as positive control. Please note that pepsin degrades all other proteins; thus, typical loading controls cannot be provided. (**F**) Immunoblotting against IBSP was performed on whole protein lysate from AC and BMSC-derived cartilage on day 35. β-actin served as loading control. Representative results are shown for samples from *n* = 3 AC and *n* = 3 BMSC donors.

To assess whether the maturation of AC- and BMSC-based neocartilage at physiologically relevant extracellular calcium levels stimulates unwanted hypertrophic side effects, the expression of hypertrophic and osteogenic markers was examined on gene and protein levels. On day 35, 8.0 mM [Ca^2+^]_e_ did not change the expression of the hypertrophic markers *COL10A1* and *MEF2C* and of the osteogenic marker *IBSP* in AC and BMSC-derived chondrocytes (Figure 4A–C). Osteogenic ALP activity remained below the detection limit under both conditions and at all time points in AC. In the BMSC group, calcium stimulation slightly increased mean ALP activity levels on day 21 (*p* = 0.31) and day 28 (*p* = 0.39), but this did not reach significance (Figure 4D). While collagen type X protein was not detectable in AC-derived neocartilage under both conditions, collagen type X levels were maintained under 8.0 mM [Ca^2+^]_e_ in samples from one BMSC donor but decreased in samples from three BMSC donors (Figure 4E and data not shown). Of note, since collagen isolation relies on pepsin digestion which degrades all other proteins, typical loading controls cannot be provided. Thus, to confirm successful collagen isolation in AC, we stained for collagen type II protein in the same lysates by cutting the blot. In line with reduced ECM formation and *COL2A1* expression, AC downregulated collagen type II levels under 8.0 mM [Ca^2+^]_e_ compared to controls. In the BMSC group, collagen type II protein was slightly elevated by 8.0 mM [Ca^2+^]_e_ and, in line with the induced GAG deposition, this confirmed improved chondrogenesis (Figure 4E). While no IBSP signal was detectable at both conditions in AC, calcium increased IBSP in samples from all BMSC donors; however, in two out of three donor samples, the signal was very faint (Figure 4F). Thus, while IBSP levels appeared slightly induced, collagen type X levels rather decreased with calcium stimulation in BMSC-derived neocartilage. Overall, long-term culture under physiologically relevant calcium levels induced no general shift to a more hypertrophic phenotype, neither in AC nor in BMSC-derived chondrocytes.

### 3.4. Unchanged Pro-Chondrogenic Signaling Activity in AC and BMSC-Derived Chondrocytes

**Figure 5:**
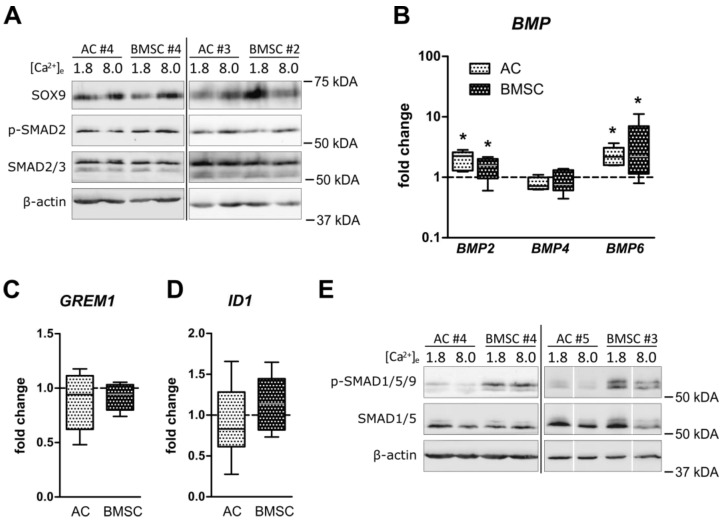
Calcium-induced regulation of SOX9 protein, TGFβ- and BMP signaling. AC- or BMSC-laden tissue engineering constructs were cultured in differentiation medium at 1.8 or 8.0 mM extracellular [Ca^2+^]_e_ for 35 days. (**A**) Immunoblotting against SOX9, phosphorylated SMAD2 and total SMAD2/3 was performed on whole cell lysates from AC and BMSC-derived chondrocytes on day 35. β-actin served as loading control. Representative blots are shown for *n* = 2–3 AC and *n* = 3- BMSC donors. (**B**–**D**) *BMP2*, *-4*, *-6, GREM1* and *ID1* mRNA levels were determined on day 35 in AC and BMSC-derived neocartilage using qPCR. Gene expression levels were normalized to the mean expression of reference genes *18S*, *GAPDH* and *RPL13* and referred to values of control samples set to one. Data are shown as box plots as indicated in Figure 1. *n* = 5 AC- and *n* = 6 BMSC donors, * *p* < 0.05, MWU, vs. 1.8 mM Ca^2+^. (**E**) Immunoblotting against total SMAD1/5 and phosphorylated SMAD1/5/9 was performed on whole cell lysates from AC and BMSC-derived chondrocytes on day 35. β-actin served as loading control. Representative blots are shown from *n* = 3 AC and *n* = 4 BMSC donors. Samples from random AC/BMSC donor pairs were always run on the same gel.

An open question was whether the calcium-induced inverse regulation of cartilage matrix formation in AC- and BMSC-based neocartilage was due to a differential regulation of prominent pro-chondrogenic pathways. Therefore, alterations in pro-chondrogenic SOX9 protein as well as TGFβ- and BMP-signaling were investigated at termination of culture at 8.0 mM [Ca^2+^]_e_ compared to controls. On day 35, Western blot analysis revealed no consistent calcium-induced changes in SOX9 protein levels in AC and BMSC-derived chondrocytes (Figure 5A). Furthermore, no change in pSmad2 as well as total Smad2/3 levels was observed after calcium stimulation in both groups, indicating that canonical TGFβ signaling was not altered (Figure 5A). Investigation of gene expression of BMP ligands *BMP2*, *-4* and *-6* upon calcium treatment identified *BMP2* and *BMP6* as calcium-sensitive genes in both cell types. No significant regulation was observed for *BMP4* (Figure 5B), the endogenous BMP inhibitor *GREM1* (Figure 5C) or the BMP response gene *ID1* (Figure 5D). Likewise, no consistent regulation of pSmad1/5/9 and total Smad1/5 levels was observed in AC and BMSC-derived chondrocytes (Figure 5E). Thus, although *BMP2* and *BMP6* mRNA expression was calcium-sensitive in both cell types, pro-chondrogenic pathway activity could not explain the inverse regulation of GAG production under elevated extracellular calcium in AC- and BMSC-based neocartilage.

### 3.5. Induction of PTHrP at Physiologically Relevant Calcium Levels in AC but Not BMSC-Derived Chondrocytes

**Figure 6:**
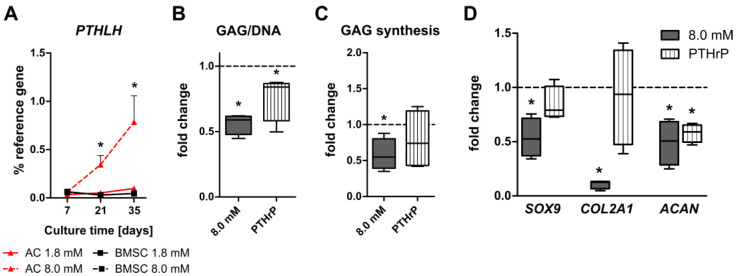
Effect of PTHrP supplementation on cartilage neogenesis. AC- or BMSC-laden tissue engineering constructs were cultured in differentiation medium for 35 days at 1.8 or 8.0 mM extracellular [Ca^2+^]_e_. (**A**) *PTHLH* mRNA levels were determined in AC and BMSC-derived chondrocytes on day 7, day 21 and day 35 using qPCR. Gene expression levels were normalized to the mean expression of reference genes *18S*, *GAPDH* and *RPL13*. Day 7 (*n* = 3), day 21 (*n* = 6) and day 35 (*n* = 7) AC donors. Day 7 (*n* = 5), day 21 (*n* = 3) and day 35 (*n* = 8) BMSC donors. Data are depicted as mean expression ± SEM. * *p* < 0.05, MWU, vs. 1.8 mM Ca^2+^. (**B**–**D**) AC-laden samples were cultured in chondrocyte differentiation medium for 35 days under control or 8.0 mM [Ca^2+^]_e_ conditions. In the PTHrP-stimulation group, constructs were treated with 10 ng/mL recombinant human PTHrP_1-34_ from day 21 to day 35. (**B**) The GAG content was measured by DMMB assay on day 35. Values were normalized to the DNA content and 1.8 mM [Ca^2+^]_e_ control samples were set to one (dashed line). (**C**) The GAG synthesis rate was measured as ^35^S-sulfate incorporation during the final 24 h of culture on day 35. Values were normalized to the DNA content and control samples were set to one. (**D**) *SOX9*, *COL2A1* and *ACAN* mRNA expression was determined by qPCR. Gene expression levels were normalized to the mean expression of reference genes *18S*, *GAPDH* and *RPL13* and referred to values of control samples set to one. Data are shown as box plots as indicated in Figure 1. *n* = 4 AC- and *n* = 4 BMSC donors. * *p* < 0.05, MWU, vs. 1.8 mM Ca^2+^.

We and others previously reported that PTHrP can have anabolic or catabolic effects on cartilage matrix formation depending on the application mode [42,43,44]. To investigate whether the calcium-sensitive PTHrP pathway may be involved in the regulation of cartilage matrix formation in calcium-treated AC- and BMSC-derived neocartilage, the expression of *PTHLH* in response to physiologically relevant calcium levels was tested. While calcium significantly upregulated *PTHLH* expression in AC on day 21 (6.6-fold, *p* = 0.026) and on day 35 (9.9-fold, *p* = 0.011), *PTHLH* expression remained low throughout BMSC chondrogenesis under all conditions (Figure 6A). A specific induction of PTHrP protein production by 8.0 mM [Ca^2+^]_e_ on day 35 was investigated for AC neocartilage lysates from three donors by an immunoradiometric assay. PTHrP protein was induced by elevated extracellular calcium in samples from two out of three AC donors. Control samples from the BMSC group in which *PTHLH* was not induced remained below the detection limit (Supplementary Table S2). This demonstrated a differential *PTHLH*-response to physiologically relevant calcium levels for AC and BMSC-derived chondrocytes inviting speculation that this may relate to the differential response in ECM formation of both cells.

In order to elucidate whether PTHrP may display a negative influence on cartilage matrix production in AC-derived neocartilage, AC constructs were exposed to exogeneous PTHrP_1-34_ (10 ng/mL). Specifically, we asked whether PTHrP_1-34_ supplementation could mimic the reduction in differentiation markers, GAG synthesis and GAG deposition in AC constructs similar to 8.0 mM [Ca^2+^]_e_. Based on results from the time course of *PTHLH* expression (Figure 6A), AC constructs were kept at control conditions for 21 days and treated from day 21 to day 35 with 10 ng/mL human recombinant PTHrP_1-34_. Parallel samples were exposed to constant 8.0 mM extracellular calcium treatment for 35 days. Similar to what was observed after calcium stimulation, the GAG/DNA ratio was significantly reduced by PTHrP_1-34_ treatment compared to controls (*p* = 0.029, Figure 6B). In addition, PTHrP_1-34_ reduced the median GAG synthesis rate in AC by 26% compared to controls, but, in contrast to calcium stimulation and due to low sample numbers, this effect did not reach significance (Figure 6C). While 8.0 mM [Ca^2+^]_e_ reduced mRNA levels of *SOX9*, *COL2A1* and *ACAN*, PTHrP_1-34_ selectively decreased the expression of *ACAN* (Figure 6D). Thus, some of the changes induced by elevated extracellular calcium in AC were also obtained under PTHrP_1-34_ treatment, indicating that the selective induction of PTHrP may contribute to the negative effects on ECM production in AC neocartilage. Further experiments to inhibit PTHrP pathway activity showed that unfortunately the known inhibitor H89 and the antagonist PTHrP_7-34_ were not able to neutralize the cyclic AMP response induced by exogenous PTHrP_1-34_, even when the inhibitor or antagonist were applied at high concentrations (data not shown). Thus, investigation of the detailed causative role of PTHrP for the calcium-induced drop of ECM production in AC-neocartilage requires more elaborative models like genetic PTHrP knock-out cell lines.

## 4. Discussion

Although several approaches exist to enhance the ECM content in neocartilage, these methods often rely on elaborate bioreactor protocols or expensive growth factor treatments. Encouraged by the positive effects of physiological oxygen on cartilage matrix formation in AC- and MSC-based tissue engineering constructs [13,45,46], we here raised extracellular calcium to physiologically relevant levels to find a simple and inexpensive means to enhance the cartilage matrix formation in engineered cartilage. Surprisingly, we recorded a differential response of AC and BMSC-derived chondrocytes to elevated extracellular calcium and were able to enhance the GAG and collagen type II formation only in BMSC-based neocartilage while ECM formation was depressed in AC. As a result, GAG/DNA values were two-fold higher in BMSC-based neocartilage under physiologically relevant calcium conditions compared to the AC group, and this success was possible without the stimulation of unwanted pro-hypertrophic side effects. Both the negative and positive effects on ECM synthesis were specific for CaCl_2_ and not due to a divalent cation effect also displayed by Mg^2+^ or by an osmotic effect displayed by sucrose. Thus, elevating extracellular calcium levels to 8.0 mM was discovered as a new simple and inexpensive means to enhance the ECM content of BMSC-based neocartilage without the need for special bioreactors. Due to the specific induction of PTHrP mRNA and protein by elevated extracellular calcium only in AC, along with similar effects obtained under recombinant PTHrP_1-34_ treatment, we postulate that the PTHrP pathway contributes to the negative outcome in AC-based neocartilage.

An important condition of this study was the investigation of calcium-induced effects on in vitro cartilage neogenesis in a clinically relevant and well characterized engineered cartilage model based on primary human AC or BMSC. We ensured that under standard chondrogenic conditions AC and BMSC-derived chondrocytes formed a GAG- and collagen type II-rich ECM that contained well-differentiated round chondrocytes. Based on this, we demonstrated for the first time an impaired GAG and collagen type II forming capacity of human AC-based neocartilage at physiologically relevant extracellular calcium levels. Negative effects on GAG deposition previously observed with juvenile bovine AC at slightly different chondrogenic conditions [22] suggest that catabolic calcium effects on AC are common between the species and rather insensitive to some culture variations.

The current work is the first to show the positive effects of elevated extracellular calcium on human MSC-based neocartilage cultured under conditions that are relevant for articular cartilage tissue engineering. Earlier studies reported reduced cartilage matrix deposition at physiologically relevant extracellular calcium levels in human BMSC [28] and human adipose tissue-derived MSC [27], but these studies were performed under unfavorable culture conditions for articular cartilage neogenesis, i.e., in the presence of pro-hypertrophic thyroxine or fetal bovine serum, containing high levels of anti-chondrogenic fibroblast growth factor. Such additives may explain the discrepancy between our results and the negative outcome in these studies [47,48]. Although the strength of the calcium effects was variable among different donor populations, the average 1.25-fold higher GAG/DNA values under elevated calcium conditions in BMSC-based neocartilage were nearly in the range of long-term mechanical loading effects (approx. 1.6-fold) [49], physioxia (approx. 1.3- to 3-fold) [13,14,45] or additional growth factor treatment (approx. 1.3- to 1.5-fold) [43,48,50,51] in previous studies using human BMSC. Thus, compared to other stimulations, treatment with physiologically relevant calcium levels provides a positive outcome regarding matrix formation at markedly reduced costs and effort.

Given that extracellular calcium concentrations around healing bone can reach up to 40 mM due to dynamic bone remodeling processes [52], this can have important implications for the design of osteochondral repair strategies where cartilage formation is desired in close vicinity to resorbable calcified structures. Under such conditions, it is important to select cells that deposit high amounts of GAG and type II collagen in the presence of elevated extracellular calcium concentrations. Although in vivo effects of calcium are currently unclear, for the clinical application of AC or MSC during osteochondral defect treatment, the choice of cells should carefully be considered in light of our data. Our study suggests the use of BMSC rather than AC for cartilage neogenesis in proximity to resorbable calcified structures such as bone or bone-mimetic replacement materials. Thus, an appealing approach for osteochondral tissue engineering is the design of multi-layered osteochondral constructs using a combination of AC and BMSC. In bizonal AC-BMSC constructs partly grown in vivo, AC-seeded non-mineralized cartilage grew on top of a BMSC-derived chondrocyte layer which developed into calcified cartilage [53] and could serve as a barrier to resorbable bone replacement structures to protect the more sensitive calcium-susceptible AC. Importantly, the benefit of a mineralized cartilage layer for cartilage defect repair in terms of tissue integration and mechanical stability was shown in several publications [54,55,56]. Furthermore, since BMSC formed more cartilage ECM in the vicinity of the calcium phosphate ceramic β-TCP in our previous work [33], the positive effects described here with extracellular calcium are also relevant for calcium-based resorbable bone replacement material applied in osteochondral tissue engineering approaches.

One important novelty of this work is that physiologically relevant extracellular calcium levels did not induce unwanted pro-hypertrophic side-effects in AC and BMSC-derived chondrocytes, and the enhanced cartilage matrix formation in BMSC-based neocartilage occurred without the expense of significantly increased hypertrophic marker expression. This was somewhat surprising considering the previously described pro-hypertrophic effects of extracellular calcium on MSC chondrogenesis but may be attributed to the pro-hypertrophic conditions in these studies [27,28]. Another surprising observation was the opposing regulation of collagen type II and collagen type X protein in BMSC-derived chondrocytes, whereas these collagens are ordinarily regulated in the same direction in MSC chondrogenesis [57,58]. Thus, the current work suggests extracellular calcium elevation as an effective approach to stimulate pro-chondrogenic collagen type II independently of pro-hypertrophic collagen type X.

Another important finding of the current work is the opposite response of AC and BMSC-derived chondrocytes to elevated extracellular calcium levels. So far, numerous differences between AC and MSC-derived chondrocytes have been described [37,57,59,60] and a differential response of AC- and BMSC-based neocartilage regarding ECM formation was also observed after mechanical stimulation [37] and in response to pro-inflammatory IL-1β or TNFα treatment [61]. The current study now provides another condition under which AC and BMSC-derived chondrocytes show a differential response to physiological stimuli. This indicates once more that isolated and expanded AC and BMSC differentiate into disparate types of chondrocytes under identical culture conditions. Interestingly, while the disparity between AC and BMSC-derived chondrocytes was disadvantageous for BMSC in the case of mechanical loading or exposure to pro-inflammatory cytokines, extracellular calcium treatment allowed BMSC-derived chondrocytes to display positive effects in contrast to AC. Investigation of the pro- and anti-chondrogenic signaling mechanism that may underly the inverse regulation of cartilage matrix production revealed a specific induction of the calcium-sensitive PTHrP pathway in AC, but not in BMSC-derived chondrocytes under physiologically relevant calcium conditions. In line with our results, previous studies have shown that extracellular calcium supplementation induces PTHrP secretion [62] and inhibits matrix production by bovine AC [22]. In the current study, for the first time we were able to show similar effects in human AC. We could also show that stimulation of AC with recombinant human PTHrP_1-34_ mimicked the high calcium-mediated reduction in *ACAN* expression and GAG/DNA deposition by AC. PTHrP_1-34_ is a truncated form of the full-length PTHrP peptide that only activates one branch of the typical PTHrP signaling response, namely signal transduction via PKA [63]. Whether specific PTHrP isoforms would be able to fully mimic the here observed calcium-induced reduction in cartilage matrix formation by AC is therefore an interesting topic for future research. Nevertheless, a recently identified point mutation in amino acid 25 of the PTHrP signaling homologue PTH (PTH^R25C^) is responsible for a lack of calcium responsiveness between PTH and its receptor [64], causing severe hypocalcemia [65]. This and the fact that similarities between PTHrP and PTH exist only within the first 34 amino acids argues in favor of the PTHrP_1-34_ peptide used here to mediate calcium-related effects. Nevertheless, our data encourage further investigations of PTHrP ablation to fully elucidate its functional role during high calcium-induced cartilage matrix destruction by AC. While this would require specific PTHrP inhibitors, known inhibitors such as PTHrP_7-34_ proved inefficient to neutralize exogeneous PTHrP_1-34_-induced signaling in our model (data not shown). In addition, while PTHrP_7-34_ specifically antagonizes PTHrP signaling via its receptor (PTH1R), previous work from our group suggested that PTHrP may exert receptor independent effects [42]. Identifying potent PTHrP inhibitors which block both the PTH1R-dependent and -independent signaling branches of the PTHrP pathway or the development of PTHrP knock out cell lines may be used in future studies to investigate whether PTHrP presents a promising target to prevent cartilage matrix destruction under physiologically relevant extracellular calcium conditions in AC.

## 5. Conclusions

We here described for the first time an inverse regulation of cartilage matrix formation by AC and BMSC-derived chondrocytes cultured at physiologically relevant extracellular calcium levels. In BMSC-based neocartilage, calcium elevation increased ECM content with a similar efficacy as more elaborate and expensive methods like mechanical loading, physioxia or additional growth factor treatment, but at markedly reduced cost and effort. Since the positive outcome of calcium on GAG synthesis was reached without unwanted pro-hypertrophic side-effects, elevated extracellular calcium is a novel, simple and inexpensive approach to enhance the cartilage matrix deposition in BMSC-derived neocartilage. In contrast, increased calcium levels should be avoided for optimal AC-derived neocartilage formation, for which the more elaborate methods remain more promising to enhance the ECM content. For application in osteochondral tissue engineering, BMSCs are the favored cell source for cartilage neogenesis in the vicinity of calcified bone replacement materials since they do not upregulate PTHrP, for which a negative influence on GAG production was observed. Further studies are now needed to unravel the exact role of the calcium-sensitive PTHrP pathway on cartilage neogenesis regarding negative effects on cartilage matrix production but positive effects on the suppression of a hypertrophic phenotype.

## Data Availability

The data presented in this study are available on reasonable request from the corresponding author.

## Supplementary Materials

The following supporting information can be downloaded at: https://www.mdpi.com/article/10.3390/cells12121659/s1, Table S1: List of qRT-PCR primers used in this study; Table S2: PTHrP protein concentration [pmol/L] in day 35 neocartilage from AC and MSC-derived chondrocytes.
