# Supplementary material for: Inverse Regulation of Cartilage Neogenesis at Physiologically Relevant Calcium Conditions by Human Articular Chondrocytes and Mesenchymal Stromal Cells
Source: Cells. 2023 Jun 18;12(12):1659. doi: 10.3390/cells12121659 (PMC10297224; doi:10.3390/cells12121659)
Supplement: Supplementary file 1 [file cells-12-01659-s001.zip › Supplement Table S1_Primer_revised.pdf]

Supplementary table S1 List of qRT-PCR primers used in this study

Gene	Fw primer 5' to 3'	Rw primer 5' to 3'
18S	GTAACCCGTTGAACCCCATT	CCATCCAATCGGTAGTAGCG
GAPDH	CCACCCATGGCAAATTCCATGGCA	TCTAGACGGCAGGTCAGGTCCACC
RPL13	CATTTCTGGCAATTTCTACAG	CAGGCAACGCATGAGGAAT
COL2A1	TGGCCTGAGACAGCATGAC	AGTGTTGGGAGCCAGATTGT
ACAN	GCACATGCCTTCTGCTT	GGAACCACTTGGGTCACG
SOX9	GTACCCGCACTTGCACAAC	TCGCTCTCGTTCAGAAGTCTC
COL10A1	TTTACGCTGAACGATACCAAA	TTGCTCTCCTCTTACTGCTAT
IBSP	CAGGGCAGTAGTGACTCATCC	TCGATTCTTCATTGTTTTCTCCT
MEF2C	GTATGGCAATCCCCGAAACT	ATCGTATTCTTGCTGCCTGG
BMP2	ACGAGGTCCTGAGCGAGTTC	GAAGCTCTGCTGAGGTGATAA
BMP4	GGATCTTTACCGGCTTCAGTC	CCTGGGATGTTCTCCAGATG
BMP6	ATTACAACAGCAGTGAATTGA	TTCATGTGTGCGTTGAGTG
ID1	ATCAGGGACCTTCAGTTGGAGC	AGACCCACAGAGCACGTAATTCC
GREM1	CCGCACTCAGCGCCAC	AGGGCTCCCACCGTGTA
PTHLH	CGGTGTTCCTGCTGAGCTA	TGCGATCAGATGGTGAAGGA
