# Supplementary material for: Inverse Regulation of Cartilage Neogenesis at Physiologically Relevant Calcium Conditions by Human Articular Chondrocytes and Mesenchymal Stromal Cells
Source: Cells. 2023 Jun 18;12(12):1659. doi: 10.3390/cells12121659 (PMC10297224; doi:10.3390/cells12121659)
Supplement: Supplementary file 1 [file cells-12-01659-s001.zip › Supplement Table S2_PTHrP protein_revised.pdf]

Supplementary table S2 PTHrP protein concentration [pmol/L] in day 35 neocartilage from AC and BMSC-derived chondrocytes

	AC			BMSC
$[Ca^{2+}]_e$	Donor #1	Donor #2	Donor #3	Donor #1
1.8 mM	<0.50	<0.50	<0.50	<0.50
8.0 mM	5,4	2,5	<0.50	<0.50
